# Factors Affecting the Antibody Immunogenicity of Vaccines against SARS-CoV-2: A Focused Review

**DOI:** 10.3390/vaccines9080869

**Published:** 2021-08-05

**Authors:** Zhangkai Jason Cheng, Mingshan Xue, Peiyan Zheng, Jiali Lyu, Zhiqing Zhan, Haisheng Hu, Yong Zhang, Xiaohua Douglas Zhang, Baoqing Sun

**Affiliations:** 1 State Key Laboratory of Respiratory Disease, National Clinical Research Center of Respiratory Disease, Department of Allergy and Clinical Immunology, Guangzhou Institute of Respiratory Health, First Affiliated Hospital of Guangzhou Medical University, Guangzhou 510623, China; jasontable@gmail.com (Z.J.C.); limorconder@gmail.com (M.X.); gdmcslxx@126.com (P.Z.); lvjialiii@163.com (J.L.); 13544377529@163.com (H.H.); zhangy@hhgene.cn (Y.Z.); 2 Department of Clinical Medicine, The Third Clinical School of Guangzhou Medical University, Guangzhou 510182, China; zhanzhiqing@foxmail.com; 3 Faculty of Health Sciences, University of Macau, Taipa, Macau 999078, China; douglaszhang@um.edu.mo

**Keywords:** COVID-19, vaccines, neutralizing antibody, variants, ADE

## Abstract

Vaccines are a crucial part of the global anti-pandemic effort against COVID-19. The effects of vaccines, as well as their common influencing factors, are the most important issues that we should focus on at this time. To this end, we review statistics on immunogenicity after vaccination, using neutralizing antibodies as the main reference index. Age, infection history, and virus variants are compared, and vaccination program recommendations are made accordingly. Suggestions are made to address concerns raised by the vaccines’ shortened development cycle, as well as the emergence of immunity escape of viral variants. Finally, a brief description and future prospects are provided based on the principle of the ADE effect and previous experience with similar viruses.

## 1. Introduction

The outbreak of SARS-CoV-2 virus infection in 2019 has resulted in a global pandemic of Coronavirus Disease 2019 (COVID-19) [1]. Virus invasion elicits an immune response, which frequently results in fever [2,3,4]. This process greatly speeds up the adhesion and migration of immune cells to lymph nodes and tissues at the site of infection. As phagocytes engulf the virus, they also release cytokines, which recruit additional immune cells to participate. Meanwhile, helper T cells stimulate B cells to produce specific antibodies that bind to the virus and prevent it from entering the cell, allowing it to aggregate and then be engulfed by phagocytes [5]. If the immune system wins such a battle against the virus, the body recovers and preserves its immunity to SARS-CoV-2, and the B cells can proliferate rapidly and produce a large number of antibodies when re-infected [6].

Antibodies can be divided into two segments, fragment crystallizable (Fc) and fragment antigen-binding (Fab), which are located on the two arms of the Y shape and can bind specific antigens with high affinity. The binding and Fc segments are conserved in the trunk of the Y shape and primarily recruit related cells, such as phagocytes, for the immune response. According to the five heavy chain types of Fc segments, γ, μ, α, ζ, and ξ, five types of immunoglobin (Ig) antibody, IgG, IgM, IgA, IgD, and IgM can be determined, respectively. IgG and IgM antibodies, in particular, play a major role in the production of antibodies by SAS-CoV-2 infection. IgG is the primary antibody against bacteria, antivirus, and antitoxin in serum. IgM is the first antibody that appears in the primary immunization [7,8], and it is the vanguard of the body’s anti-infection mechanism [9]. Furthermore, since the glycoprotein on the viral spike protein mediates entry into human cells via the ACE2 receptor, anti-spike neutralizing antibodies (nAbs) can block the viral infection of human cells and counter viral replication.

In the absence of targeted treatment, the best way to control the number of infections and protect healthy people is to establish universal immunity through vaccination. Vaccines can stimulate the body’s immune system to recognize the virus as a threat, and protect against infection via both antibody and cellular immunity. To this end, countries around the world are speeding up the progress of vaccine research. There is a multitude of factors that can influence the immune response to vaccination, including intrinsic host factors, extrinsic factors, environmental factors, behavioral factors, nutritional factors, vaccine factors, and administration factors [10]. Although the neutralization test can be used as a direct indicator of vaccine efficacy, cellular immunity also plays an important role [11]. According to the statistics of Plotkin et al. [12], the CD4 + response is essential for helper B cell and cytokine production and is sometimes more associated with protection than with antibody titers. This review, however, will only cover antibody immunity.

In this review, we examine several common influencing factors on vaccines’ ability to elicit antibody production and discuss the potential and future strategies of vaccines. After thoroughly reviewing articles in relevant fields, we selectively present some of them, and summarize, analyze, and extend the results as follows.

## 2. Vaccination

The goal of SARS-CoV-2 vaccines is to produce nAbs that recognize the viral S protein. Such anti-spike nAbs can prevent virus–human cell interaction and aid in the elimination of infection in its early stages. Vaccines differ in terms of their mechanism of action, mode of administration, and type of immune response. As a result, the relevant immunological evaluation indexes, such as nAb level and T cell immune response intensity, have been disclosed in animal and clinical trials centered on the core points of their vaccines [13,14,15]. The mechanism by which SARS-CoV-2 interacts with the immune response is still unknown, which influences the selection of key indexes for vaccine efficacy evaluation, with nAb immunity being the most important protective immune index. In healthy adults, antibody responses to SARS-CoV-2 peak at day 28 after vaccination [16], and rapid specific T-cell responses have been observed as early as day 14 [17]. The study of the protective effect, on the other hand, is still ongoing. Continuous observational studies are required to determine whether a sustained protective effect can be achieved, how long antibodies can provide a sustained protective effect, and whether vaccination should be strengthened [18]. Long-term immunity must be monitored. We compared and analyzed age differences in the vaccine’s effects, the antibody level produced by the vaccine in people with and without a history of infection, the influence of a previous infection on vaccine effect, and the effect of the vaccine on mutated viruses.

### 2.1. Age Factor

Frenck Jr. et al. [19] conducted experiments and statistics on the antibody production results of vaccinated adolescents aged 12 to 17 years. The immune responses to BNT162B2 in adolescents aged 12 to 15 years (ID50 geometric-mean-titer (GMT) of 1283.0) were higher than those observed in adolescents aged 16 to 25 years (GMT of 730.8) [19]. In Ramasamy et al.’s study [20], the nAb titer peaked 42 days after the second vaccination of ChAdOx1, and the neutralization titer did not differ significantly between the vaccinated population aged 18 to 55 years and those over 55 years of age. In Xia et al.’s study on the BBIBP-CorV vaccine [21], people aged 60 and above produced significantly fewer nAbs than those aged 18–59. Doria-Rose et al. [22] examined the presence of long-term antibodies 180 days after the second vaccination of mRNA-1273. In the live virus attenuation and mNeonGreen tests, all age groups demonstrated antibody activity. For age groups 18 to 55, 56 to 70, and 71 or older, the ID50 GMT was 406, 171, and 131, respectively. Müller et al.’s study [11] showed that 31.3% of those aged 80 and older who received the BNT162b2 vaccine still had no nAbs after the second dose, compared to 2.2% of those younger than 60. Dr. Bubar used mathematical modeling to stratify five groups by age and to discuss the impact of a given group on overall infection rates, mortality rates, and other factors. A highly effective transmissible blocking vaccine given to adults aged 20 to 49 that would minimize cumulative morbidity, while giving priority to adults aged 60 and older would have the lowest mortality rate [23]. Buckner et al.’s model yielded comparable results [24]. Table 1 summarizes vaccine studies that compare nAb titers across different age groups. The findings indicated that vaccination could benefit people of all ages, but that younger people should be prioritized in cases where vaccination is insufficient.

### 2.2. Infection History

Turner et al. [28] recently found that while the nAb levels of previously infected individuals declined rapidly in the fourth month, long-lived bone marrow plasma cells could be detected to have a long-term ability to resist SARS-CoV-2, demonstrating that individuals with past infection history have the ability to maintain a certain level of antibody. Comparing recovered patients to vaccinated healthy individuals, Anderson et al. [29] used three live virus neutralization methods to analyze antibodies 14 days after the second vaccination of mRNA-1273 and found that all participants had effective neutralization responses. GMT of participants in the 100 μg vaccinated subgroup significantly exceeded that of participants who had recovered from previous infections. The binding antibody and nAb responses in the vaccine group appeared to be similar to those previously reported in vaccinators aged 18 and 55 years old and were higher than the median in the recovered patient group [29]. As a result, previously infected patients have nAbs, but not at the same level as having the vaccine, so previously infected patients who want higher levels of effective antibodies must also be vaccinated.

Whether previously infected people need to receive the same full range of vaccinations as those who are not infected is up for debate. Anichini et al. [30] compared data on the difference between vaccination regimens using the BNT162b2 vaccine, for patients with previous COVID-19 infection and those without previous COVID-19 infection, to determine whether different vaccination regimens are required for patients with previous COVID-19 infection and those without previous COVID-19 infection. The presence of specific anti-SARS-CoV-2 nAbs in the serum samples was investigated. GMT of previously infected subjects was 569, significantly higher than 118 for previously uninfected subjects. Furthermore, nAb titers were significantly lower in uninfected patients who received the second dose of vaccine than in previously infected subjects who received only one dose of vaccine [30]. Therefore, in areas where vaccine production is not sufficient for the entire course, it may be considered to give patients previously infected with COVID-19 only a single dose of vaccine to maximize the vaccine’s effect.

### 2.3. Virus Mutation

The emergence of SARS-CoV-2 variants has raised concerns that these variants may evade immunity resulting from prior infection or vaccination. A list of major mutation variants is provided by the World Health Organization (WHO) [31]. Wang et al. [32] compared the neutralizing activity of convalescent serum and vaccinee serum against D614G, B.1.1.7, and B.1.351 pseudovirus variants to wild-type pseudovirus. According to the findings, B.1.1.7 showed little resistance to the neutralizing activity of convalescent or vaccinee serum, whereas B.1.351 showed more resistance to both convalescent serum neutralization (by a factor of two) and vaccinee serum neutralization (by a factor of 2.5 to 3.3) than the wild-type virus. The majority of the tested vaccinee serum samples lost neutralizing activity, which was consistent with the findings of other recent studies of neutralization by convalescent serum or serum obtained from recipients of mRNA or BBIBP-CorV vaccines [33,34,35]. Diamond et al. [36] used human sera from recipients of the Pfizer-BioNTech (BNT162b2) mRNA vaccine, and nearly all mRNA vaccine-induced immune sera tested had significantly reduced neutralizing activity against the South African (Wash SA-B.1.351) strain or recombinant viruses. The findings emphasize the importance of ongoing viral monitoring and evaluation of vaccines’ protective efficacy in areas where variants are circulating. Wong et al. [32] investigated the inhibitory effect of vaccine-produced antibodies against the two variants. B.1.1.7 demonstrated little resistance to the neutralization activity of convalescent or vaccinated sera, whereas B.1.351 demonstrated strong resistance to both convalescence and vaccinated sera. Dr. Madhi’s [37], Dr. Chen’s [38], Dr. Wu’s [39], and Dr. Shinde’s [40] experiments yielded similar results. Wall et al. [41] found the B.1.617.2 strain to have very strong resistance to vaccinated sera, even more so than B1.351. Edara et al. [42] also reported a strong resistance. Results from studies investigating the neutralization effects of vaccines against major variants are compared in Table 2. Although the trial results are optimistic for the resistance of the mutant virus, two recent cases of immunization escape of the SARS-CoV-2 variant against the vaccine have been reported, with mutations including E484K and D614G in Patient 1. In Patient 2, mutations included D614G and S477N. Hacisuleyman et al. [43] tested the serum samples obtained from Patient 1 against the wild-type virus, the E484K mutant, and the B.1.526 variant, and found that the serum was equally effective against each virus. These findings suggest that while the antibody response in Patient 1 recognizes these variants, it is insufficient to prevent breakthrough infections at a high viral load. On the one hand, this serves as a reminder not to underestimate virus mutation and the possibility of escape brought by high viral load. On the other hand, it also indicates the direction of the mutation that needs to be paid attention to. To maintain the epidemic prevention achievements at this stage and prevent the recurrence of the epidemic, vigilance and awareness of the mutated virus should be maintained at all times, and the neutralization and activity of the newly discovered mutated strain should be detected in time.

## 3. Challenges and Potential Strategies for Future

The development process of new vaccines has been compressed from 10–15 years to 1–2 years [47]. They have been designed by a large number of researchers, undergoing animal experiments and large-scale clinical trials involving tens of thousands of people in the first, second, and third phases. The first two mRNA vaccines to hit the U.S. market took only 10 months from development to use, but no necessary steps were skipped in the development process. This rapid pace is due to the knowledge and experience gained during previous epidemics, the maturity of advanced technology platforms, and the timely and large-scale investment of enterprises, governments, and non-profit organizations without cost concern. This enables development and mass production to proceed simultaneously, reducing the time spent waiting for funding, waiting for sufficient subjects, and waiting for trial approval.

The global pandemic allowed phase III clinical trials to produce enough positive cases in as little as three months to analyze the vaccine’s efficacy. For other infectious diseases that are not widespread, it can take years to obtain results from clinical trials. No new drug in the world has ever had so many data from clinical trials at the time of its creation, let alone a huge amount of real-world data once the vaccine is on the market. It also benefited from the FDA’s Emergency Use Authorization (EUA) mechanism and its timely establishment of clear safety requirements and effectiveness thresholds, as well as the full process of supervision and guidance and timely review efforts.

Although the clinical trial results of the approved vaccines have been made public, the efficacy (effects in a test population under strictly defined clinical trial conditions) and effectiveness (effects in real-world populations) of the various vaccines cannot be compared due to the lack of parallel studies. Furthermore, we must consider not only routine safety and efficacy when developing nAb-based treatments and vaccines, but also issues such as escape mutations and antibody-dependent enhancement (ADE).

### 3.1. Escape Mutations of SARS-CoV-2

SARS-CoV-2 is an RNA virus with a high rate of surface protein amino acid mutation. Many SARS-CoV-2 mutants have been discovered to date, and some mutants have demonstrated resistance to nAbs [48]. Recently, cases of escape mutations have been reported [43]. Thus far, the D614G mutation of SARS-CoV-2 S protein is considered to be the main mutation detected, which may lead to an increase in infectivity and mortality [48,49]. Although the potency of RBD-targeting nAbs against the D614G variant was not reduced, and antibodies stimulated by natural infection with SARS-CoV-2 containing D614 or G614 can be cross-neutralized, the conformational transfer induced by D614G towards ACE2 binding active states may still affect the effectiveness of some nAbs (e.g., type III nAbs that only bind closed RBDs) [50]. On the other hand, the virus with any of the mutations on E484, F490, Q493, and S494 of the S protein shows complete or partial resistance to the potent nAbs (C121 and C144) [51]. Aside from natural mutations, antibody therapy and vaccine selection pressure may also play a role in mutation screening and enrichment. As a result, before we can better understand the role of D614G in natural SARS-CoV-2 infection, any vaccine or treatment design should take the presence and potential impact of the mutation into account.

To deal with the emergence, the following strategies may be effective means for antibody treatment to suppress the escape variants.

- Combination treatment of two or more nAbs (cocktail)-targeting different epitopes. It was found that the combination of multiple antibodies, especially the combination of antibodies targeting different epitopes, could reduce the mutation frequency and prevent immune escape [52]. Similarly, compared with single mAb treatment, mixed antibodies (C121 + c135 or c144 + c135) significantly reduced the emergence of drug-resistant strains. Recent studies [53,54] have found that mixing vaccines leads to good immunogenicity results, which is a promising step in this direction.
- Different live virus mutants can be used to identify antibody neutralization tests in vitro to determine the virus neutralization spectrum of antibodies.
- The development of high-neutralizing-ability antibodies against conserved epitopes is also an effective strategy for preventing immune escape caused by virus mutation.

### 3.2. ADE Consideration and Fc Engineering 

When pathogens enter the body, the viruses are captured by the immune system, and antibodies are produced in response, but antibodies sometimes amplify the damage that the virus can cause to the body, known as antibody-dependent enhancement (ADE). This is a phenomenon in which non-neutralizing or sub-neutralizing antibodies promote virus infection and lead to more serious diseases. To date, the Dengue virus, SARS-CoV, MERS-CoV, Ebola virus, and influenza virus have all been linked to ADEs [55]. Although no relevant research results from around the world have been published to confirm that SARS-CoV-2 causes ADE, it is speculated to be possible. The effects of ADE are not easily simulated or observed in an in vitro system because the protective and potentially harmful antibody-mediated mechanisms are the same [56], which also affects the study. Previous studies have shown that ADE has multiple mechanisms [57,58]. The effect of ADE mediated by Fc receptor (FCR) is mainly through the interaction between the Fc fragment of antibody and FCR on the cell surface, which makes the virus and antibody complex combine with cells with FCR, such as macrophages, monocytes, B cells, neutrophils, and granulocytes, causing the virus to adhere to the cell surface and promote infection. In the complement-receptor-mediated ADE effect, viral surface proteins can activate the classical complement system pathway by binding antibodies of different serotypes. Complement C1q binds to the protein on the virus’s surface, causing the virus to combine with the cell by binding to the C1q receptor on the cell surface, resulting in the virus infecting the cell. Previous studies have also reported a variety of ADE mechanisms, such as the ADE effect mediated by virus surface protein and the ADE effect of antibody-mimicking cell receptor binding to the virus [59,60,61]. Previous clinical studies [60] on coronavirus have shown that when SARS-CoV bound with antibody binds to FcγRII of human macrophages, it activates related downstream signals. Flores et al. [62] proved that ADE occurs on those highly effective nAbs against MERS-CoV RBD, which means that it may also occur on nAbs against SARS-CoV-2. It is believed that the generation of the ADE effect can be effectively avoided by immune focusing—developing vaccines capable of inducing high titer of nAbs and lower levels of non-neutralizing antibodies in vivo [63]. It has also been reported [64] that in the design of an HIV vaccine, removing the infection-enhancing epitope while keeping the neutralizing epitope can result in better immune protection.

In order to avoid the ADE effect and reduce the risk of Fc-mediated acute lung injury, Shi et al. [65] introduced the LALA mutation in the Fc segment of antibody CB6 (CB6-LALA). Winkler et al. [66] recently proposed that although Fc effector functions are dispensable when representative neutralizing mAbs are administered as prophylaxis, they are necessary for optimal protection when they are used as therapeutic drugs. When administered after infection, the intact mAbs reduced the SARS-CoV-2 burden and lung disease of mice and hamsters better than loss-of-function Fc variant mAbs. Thus, potently neutralizing mAbs utilize Fc effector functions during therapy to mitigate lung infection and disease. Li et al. [67] reported the first in vivo evidence on the ADE effect of SARS-CoV-2, finding that even direct injection of mAbs with a strong ADE effect in vitro did not result in significant in vivo effects such as increased viral load, inflammation, and so on. On the contrary, it has a partial protective effect. There could be two reasons for this: first, SARS-CoV-2 cannot replicate effectively in macrophages, whereas Fc-mediated ADE antibody primarily enters macrophages; second, Fc-mediated effector function plays a protective role. At the same time, a recent study by Liu et al. [68] found that some antibodies found in patients specific to the S protein cannot neutralize the virus, but when they bind to the S protein, they can change its conformation and enhance ACE2 receptor binding. This enhancing antibody does not work when the nAb level is high. However, when the nAb levels are still low in the pre-infection or early infection stage, the enhancing antibody levels may affect the progression of COVID-19, prolonging the course of the disease and exacerbating symptoms.

Although current evidence does not support that SARS-CoV-2 infection or vaccination can cause obvious ADE effects, the ongoing COVID-19 vaccine efficacy testing should be monitored for the possibility of vaccine-related intensification diseases when suboptimal nAb titers are induced. It may be helpful to select candidate vaccines that elicit strong nAbs and Th1-dominant responses, balanced CD4/CD8, and multifunctional T-cell responses (while avoiding vaccines that cause Th2-dominant responses and non-neutralizing antibodies).

## 4. Conclusions

Three common factors influencing vaccine immunogenicity were reviewed and discussed. Based on the current emergence of more and more viral mutation variants, we must pay close attention to whether vaccines are effective for newly emerged, particularly multiple mutated strains, and whether they will cause the ADE effect.

## Figures and Tables

**Table 1:** Comparison of vaccinee serum 50% neutralization GMT across different age groups. It should be noted that GMT values are qualitative indicators of neutralization capability, and value comparison should be made with care.

Study	Vaccine	Type	Dose	Median/Mean Age (Age Range)	N	nAb	Days Since Last Injection	Type
						GMT		
Wu [25]	CoronaVac	Inactivated vaccine	2 × 1.5 μg	(60–64)	38	26.5	28	authentic virus neutralization test
			2 × 3 μg	(60–64)	39	36.4	28	
			2 × 6 μg	(60–64)	39	55.2	28	
			2 × 1.5 μg	(65–69)	35	21.1	28	
			2 × 3 μg	(65–69)	33	44.5	28	
			2 × 6 μg	(65–69)	40	50.4	28	
			2 × 1.5 μg	(≥70)	27	22.7	28	
			2 × 3 μg	(≥70)	28	48.2	28	
			2 × 6 μg	(≥70)	20	40.2	28	
Xia [21]	BBIBP-CorV	Inactivated vaccine	2 × 2 μg	42.7 (18–59)	32	87.7	14	
			2 × 4 μg	37.7 (18–59)	32	211.2	14	
			2 × 8 μg	40.1 (18–59)	32	228.7	14	
			2 × 2 μg	65.9 (≥60)	32	80.7	14	
			2 × 4 μg	67.5 (≥60)	32	131.5	14	
			2 × 8 μg	67.5 (≥60)	32	170.9	14	
Ramasamy [20]	ChAdOx1 nCoV-19	Adenoviral vector vaccine	2 × 2.2 × 10^10^ virus particles	44.5 (18–55)	41	161	14	
			2 × (3.5–6.5) × 10^10^ virus particles	39.0 (18–55)	39	193	14	
			2 × 2.2 × 10^10^ virus particles	60.4 (56–69)	28	143	14	
			2 × (3.5–6.5) × 10^10^ virus particles	59.5 (56–69)	20	144	14	
			2 × 2.2 × 10^10^ virus particles	73.5 (≥70)	34	150	14	
			2 × (3.5–6.5) × 10^10^ virus particles	74.0 (≥70)	47	161	14	
Sadoff [26]	Ad26.COV2.S	Recombinant adenovirus vaccine	5 × 10^10^ viral particles	36.1 (18–55)	162	224	29 after first dose	
			1 × 10^11^ viral particles	34.8 (18–55)	158	354	29 after first dose	
			5 × 10^10^ viral particles	69.6 (≥65)	161	277	29 after first dose	
			1 × 10^11^ viral particles	70.0 (≥65)	161	212	29 after first dose	
Rose [22]	mRNA-1273	mRNA vaccine	2 × 100 μg	(18–55)	14	1388	14	pseudotype neutralization test
			2 × 100 μg	(18–55)	15	775	90	
			2 × 100 μg	(18–55)	15	406	180	
			2 × 100 μg	(56–70)	9	1425	14	
			2 × 100 μg	(56–70)	9	685	90	
			2 × 100 μg	(56–70)	9	171	180	
			2 × 100 μg	(≥70)	10	900	14	
			2 × 100 μg	(≥70)	10	552	90	
			2 × 100 μg	(≥70)	9	131	180	
Walsh [27]	BNT162b1	mRNA vaccine	10 µg	26.5 (18–55)	12	180	14	
			20 µg	49.0 (18–55)	12	203	14	
			30 µg	33.5 (18–55)	12	437	14	
			10 µg	68.5 (65–85)	12	97	14	
			20 µg	69.0 (65–85)	12	292	14	
			30 µg	69.0 (65–85)	12	163	14	
	BNT162b2	mRNA vaccine	10 µg	37.0 (18–55)	12	33	14	
			20 µg	38.0 (18–55)	12	105	14	
			30 µg	36.5 (18–55)	12	105	14	
			10 µg	67.0 (65–85)	12	111	14	
			20 µg	68.5 (65–85)	12	81	14	
			30 µg	68.0 (65–85)	12	206	14	
Frenck Jr [19]	BNT162B2	mRNA vaccine	2 × 30 μg	14.0 (12–15)	1131	1283	30	
			2 × 30 μg	18.0 (16–25)	537	730.8	30	

**Table 2:** Vaccinee serum 50% neutralization GMT against variant viruses.

Study	Vaccine	Variant	N	nAb GMT	Type of Neutralization Test
Edara [44]	mRNA-1273	A.1	14	186	authentic virus neutralization test
		B.1	14	110	
		B.1.1.7	14	116	
		N501Y	14	141	
Huang [45]	BBIBP-CorV	B1.1.7	12	71.5	
	ZF2001	B1.1.7	12	66.6	
Liu [34]	BNT162b2	B.1.1.7	20	663	
		P.1	20	437	
		B.1.351	20	194	
		B.1.351 − Δ242-244 + D614G	20	485	
		B.1.351 − RBD + D614G	20	331	
Wall [41]	BNT162b2	B.1.617.2	250	0.17 *	
		B.1.1.7	250	0.38 *	
		B.1.351	250	0.20 *	
Edara [42]	mRNA-1273	A.1	15	1332	
		B.1.617.2	15	190	
	BNT162b2	A.1	10	1176	
		B.1.617.2	10	164	
Wang [32]	BBIBP-CorV	D614G	25	1.7 *	
		B1.1.7	25	1.4 *	
		B1.1351	25	0.4 *	
	CoronaVac	D614G	25	0.8 *	
		B1.1.7	25	0.5 *	
		B1.1351	25	0.3 *	
Wang [46]	mRNA-1273	B.1.351	12	0.09 *	pseudotype neutralization test
		B.1.1.7	12	1.5 *	
	BNT162b2	B.1.351	10	0.11 *	
		B.1.1.7	10	1.2 *	
Madhi [46]	ChAdOx1	B.1.351	25	74	
		Triple-mutant pseudovirus	25	85	
Chen [42]	CoronaVac	D614G	93	42.4	
		B1.1.7	93	34	
		B1.429	93	41	
		P.1	93	13.1	
		B1.526	93	12.7	
		B1.351	93	9.7	

* Fold-change relative to the wild-type virus.

## Data Availability

All data reported in this study can be found in the literature and are referenced.
